# The soluble receptor for advanced glycation end products is potentially predictive of pulmonary arterial hypertension in systemic sclerosis

**DOI:** 10.3389/fimmu.2023.1189257

**Published:** 2023-06-20

**Authors:** Isabella M. Atzeni, Yehya Al-Adwi, Berber Doornbos-van der Meer, Caroline Roozendaal, Alja Stel, Harry van Goor, C. Tji Gan, Michael Dickinson, Wim Timens, Andries J. Smit, Johanna Westra, Douwe J. Mulder

**Affiliations:** ^1^ Department of Internal Medicine, Division of Vascular Medicine, University Medical Centre Groningen, University of Groningen, Groningen, Netherlands; ^2^ Department of Rheumatology and Clinical Immunology, University Medical Centre Groningen, University of Groningen, Groningen, Netherlands; ^3^ Department of Laboratory Medicine, University Medical Centre Groningen, University of Groningen, Groningen, Netherlands; ^4^ Department of Pathology and Medical Biology, University Medical Centre Groningen, University of Groningen, Groningen, Netherlands; ^5^ Department of Pulmonary Diseases and Tuberculosis, University Medical Centre Groningen, University of Groningen, Groningen, Netherlands; ^6^ Department of Cardiology, University Medical Centre Groningen, University of Groningen, Groningen, Netherlands

**Keywords:** PAH, sRAGE, predictability, SSc, scleroderma, ILD

## Abstract

**Introduction:**

Pulmonary arterial hypertension (PAH) and interstitial lung disease (ILD) are the leading causes of death in systemic sclerosis (SSc). Until now, no prospective biomarker to predict new onset of SSc-ILD or SSc-PAH in patients with SSc has reached clinical application. In homeostasis, the receptor for advanced glycation end products (RAGE) is expressed in lung tissue and involved in cell-matrix adhesion, proliferation and migration of alveolar epithelial cells, and remodeling of the pulmonary vasculature. Several studies have shown that sRAGE levels in serum and pulmonary tissue vary according to the type of lung-related complication. Therefore, we investigated levels of soluble RAGE (sRAGE) and its ligand high mobility group box 1 (HMGB1) in SSc and their abilities to predict SSc-related pulmonary complications.

**Methods:**

One hundred eighty-eight SSc patients were followed retrospectively for the development of ILD, PAH, and mortality for 8 years. Levels of sRAGE and HMGB1 were measured in serum by ELISA. Kaplan-Meier survival curves were performed to predict lung events and mortality and event rates were compared with a log-rank test. Multiple linear regression analysis was performed to examine the association between sRAGE and important clinical determinants.

**Results:**

At baseline, levels of sRAGE were significantly higher in SSc-PAH-patients (median 4099.0 pg/ml [936.3-6365.3], p = 0.011) and lower in SSc-ILD-patients (735.0 pg/ml [IQR 525.5-1988.5], p = 0.001) compared to SSc patients without pulmonary involvement (1444.5 pg/ml [966.8-2276.0]). Levels of HMGB1 were not different between groups. After adjusting for age, gender, ILD, chronic obstructive pulmonary disease, anti-centromere antibodies, the presence of puffy fingers or sclerodactyly, use of immunosuppression, antifibrotic therapy, or glucocorticoids, and use of vasodilators, higher sRAGE levels remained independently associated with PAH. After a median follow-up of 50 months (25-81) of patients without pulmonary involvement, baseline sRAGE levels in the highest quartile were predictive of development of PAH (log-rank p = 0.01) and of PAH-related mortality (p = 0.001).

**Conclusions:**

High systemic sRAGE at baseline might be used as a prospective biomarker for patients with SSc at high risk to develop new onset of PAH. Moreover, high sRAGE levels could predict lower survival rates due to PAH in patients with SSc.

## Introduction

Interstitial lung disease (ILD) and pulmonary arterial hypertension (PAH) are the leading causes of death in systemic sclerosis (SSc) (1), a heterogeneous disease characterized by vasculopathy and immune activation, eventually leading to irreversible skin and internal organ fibrosis. Currently, no disease-modifying therapies are showing satisfactory results. Therefore, identifying disease markers that might help to predict the course of the disease is very useful. Some biomarkers have been identified for diagnosis, prognosis, and response to treatment in SSc-ILD (2). Other biomarkers were linked to SSc-PAH (3, 4),. However, no reliable prospective biomarker has been identified that can predict new onset of SSc-ILD or SSc-PAH in patients with SSc.

RAGE, a multi-ligand cell surface receptor that is expressed on a wide range of cell types, including fibroblasts, endothelial cells, and smooth muscle cells, has been implicated in numerous diseases (5, 6). Besides being the most important receptor for advanced glycation end products (AGEs), RAGE can bind exogenous and endogenous molecules, such as danger-associated molecular patterns (DAMPs) including S100A8, S100A9 and S100A12/calgranulin and high mobility group box 1 (HMGB1), leading to inflammation through NF-kB activation (7). Its soluble form (sRAGE) is established by alternative splicing of RAGE mRNA, or proteolytic cleavage of the membrane-bound form (mRAGE) by Matrix Metalloproteinase 9 (MMP9) or A-Disintegrin and Metalloprotease 10 (ADAM10) (8, 9). Although sRAGE may act as a decoy receptor for RAGE ligands which could lead to tissue damage (10), its exact role is still not completely clear.

RAGE is ubiquitously expressed in almost all tissues at relatively low levels. However, it is highly expressed in healthy lung tissue, especially on the alveolar type I cells, suggesting that RAGE may have a significant role in lung homeostasis, particularly in cell spreading and growth (11). While RAGE overexpression has been demonstrated in the fibrotic conditions in different organ systems, mRAGE and sRAGE expressions are strongly decreased in blood and lung tissue of patients with idiopathic pulmonary fibrosis (IPF) (11, 12). Also, in airway diseases such as chronic obstructive pulmonary disease (COPD) decreased sRAGE levels are described (12). In contrast, upregulation of (s)RAGE has been shown in serum and lung tissue of patients with PAH compared to healthy controls, giving rise to extracellular matrix accumulation, pulmonary artery smooth muscle cell proliferation, and apoptosis resistance (13, 14).

A potential role for HMGB1 has also been described in PAH development (14). HMGB1 is known to be released extracellularly in response to cellular injury or stress and functions as a DAMP that signals, as mentioned, through RAGE but also through the toll-like receptors (TLR4 and TLR2). The downstream processes of the HMGB1-RAGE/TLR4 axis are pro-inflammatory (15). Its serum and lung tissue levels have been reported to be increased in PAH patients and serum levels correlated with mean pulmonary arterial pressure (mPAP) (16). Similarly, serum HMGB1 is reported to be elevated in patients with SSc-ILD compared to SSc patients without ILD and healthy controls (17).

Thus, we hypothesized that high levels of sRAGE and/or HMGB1 would predict new onset of PAH and PAH-related mortality in patients with SSc while low levels would predict ILD. This may enable early identification of these devastating complications, and potentially set the stage for early intervention strategies in future studies.

## Materials and methods

### Patients

In the current study, a total of 188 SSc patients were included from 2013 to 2020. Patients were classified according to ACR/EULAR criteria for SSc (18). Demographic and disease data were gathered retrospectively by screening medical records. Also, the use of medication was collected and divided into three groups: immunosuppression or antifibrotic therapy, glucocorticoids, and vasodilators. Pulmonary function tests (PFTs) and blood were collected from routine clinical care through our outpatient clinic. The date of first blood sampling was considered as baseline and all serum was stored at -20°C. PFTs were following ATS/ERS guidelines and were defined by diffusing capacity for carbon monoxide (DLCO), forced vital capacity (FVC), or forced expiratory volume in 1 second (FEV1). An abnormal DLCO was defined when the predicted value was < 80% and an abnormal FVC when the predicted value was < 70%. The FEV1/FVC ratio and medical history were used for the diagnosis of COPD. ILD was diagnosed by PFTs and high-resolution computed tomography (HRCT) using the diagnostic algorithm according to the British Thoracic Society guidelines (19). Patients underwent a right heart catheterization if echocardiography showed tricuspid regurgitant velocity > 2.8 and/or typical echocardiographic features of PH. PAH was diagnosed based on pulmonary arterial pressure ≥ 25 mmHg and pulmonary artery wedge pressure ≤ 15 mmHg at rest measured by right heart catheterization according to the ESC/ERS 2015 guidelines (20). Other pre-capillary causes of PH were excluded using a standardized work-up and discussion in a multidisciplinary expert team. Development of ILD, PAH, and mortality were assessed during follow-up, adjudicated by two investigators, reaching a consensus diagnosis. When a consensus diagnosis was not reached, patients were discussed in multidisciplinary consultations and a final diagnosis was concluded. According to Dutch law and University Medical Centre Groningen regulations, this type of study did not fall within the scope of Medical Research Involving Human Subjects Act. The local ethics committee provided approval with exemption from written informed consent. This study was registered in the research register of the University Medical Centre Groningen (201900260).

### Enzyme-linked immunosorbent assays

HMGB1 and sRAGE serum concentrations were measured by enzyme-linked immunosorbent assays (ELISAs). HMGB1 was quantified using a commercial ELISA kit (TECAN, the Netherlands) while sRAGE was measured using a Duoset kit (R&D systems, United Kingdom), according to manufacturers’ instructions. High-performance ELISA buffer (Sanquin, the Netherlands) was used during serum incubation to prevent non-specific reactions. ELISAs were read using a microplate reader at 450-575nm and analyzed using Softmax software.

### Immunohistochemistry

For a pilot study, formalin-fixed paraffin-embedded human lung tissues obtained from two SSc-PAH patients were used for immunohistochemistry studies. Briefly, the lung tissues were cut at 3 microns sections, mounted on glass slides, and deparaffinized. For RAGE immunohistochemistry, antigen retrieval was performed with tris-HCl and EDTA. Mouse-anti-human RAGE (Santa Cruz, (A-9): sc-365154, Dallas, Texas, USA) was added overnight. Consecutively, samples were incubated with rabbit anti-mouse-HRP conjugate (Dako, 0260, Santa Clara, USA), and stained with diaminobenzidine as a substrate (Dako K4006, Santa Clara, USA). Negative control staining was performed by omission of RAGE antibody showing no aspecific binding. For determining RAGE localization, additional staining was performed on serial sections as follows. For comparison, standard histochemical Haematoxylin-Eosin and Verhoeff’s elastin stains were performed, in addition to, immunohistochemical stains for CD31 (endothelial cells), alpha-smooth muscle actin ([α-SMA] myofibroblasts and smooth muscle cells), and desmin (smooth muscle cells). The latter immunohistochemical stains were performed using an automated Ventana Benchmark Ultra immunostainer (Roche Diagnostics Netherlands) in our ISO15189 accredited pathology department.

### Statistical analysis

Statistical analysis was performed using IBM SPSS Statistics version 23. Patients were divided based on pulmonary involvement into four groups: no history of pulmonary involvement, ILD, PAH, or ILD and PAH. Data are presented as median and interquartile range (IQR) or as number (%). The Kruskal Wallis test and Mann-Whitney U test were used for comparison between groups. Spearman correlation coefficients were performed to measure associations. Kaplan-Meier survival curves were performed to predict lung events and mortality, and event rates were compared with log-rank test. Univariate analysis was used to identify factors potentially associated with PAH. In addition to age and gender, variables with significant associations were then used in the multivariate model. Multiple linear regression analysis was performed to examine the association between sRAGE and important clinical determinants at baseline. In our first model, we adjusted for age, gender, and PAH. Second, we added ILD and COPD. Third, anti-centromere antibody (ACA) positivity was added. Fourth, we added the presence of puffy fingers or sclerodactyly, and, finally, use of immunosuppression, antifibrotic therapy, glucocorticoids, or vasodilators were added. P-values < 0.05 were considered significant.

## Results

### Patient characteristics

The patient characteristics are shown in **Table 1**. At baseline, of the 188 included patients, 124 had no history of pulmonary involvement, 41 had ILD, 12 had PAH, and 11 had a history of both ILD and PAH. In all subgroups, the limited cutaneous form of SSc was most prevalent. As expected, the percentage of predicted FVC and DLCO was lower in ILD- and PAH- patients compared to SSc patients without pulmonary involvement. Moreover, treatment with immunosuppression was most often seen in ILD- and ILD+PAH- patients, while vasodilators were more prevalent in PAH- and PAH+ILD- patients.

**Table 1:** Patient characteristics at baseline.

	SSc patients without pulmonary involvement (n=124)	SSc patients with ILD (n=41)	SSc patients with PAH (n=12)	SSc patients with ILD-PAH (n=11)	Kruskal Wallis Test
Age in years, median (IQR)	61 (54–72)	65 (53-72)	70 (66-78)	64 (58-72)	p = 0.102
Female, n (%)	102 (82.3)	28 (68.3)	9 (75.0)	6 (54.5)	p = 0.077
Extent of skin involvement, n (%)					p = 0.188
Limited Diffuse Other	114 (91.9) 9 (7.3) 1 (0.8)	35 (85.4) 6 (14.6) 0 (0.0)	10 (83.3) 2 (16.7) 0 (0.0)	8 (72.7) 3 (27.3) 0 (0.0)	
Puffy fingers or sclerodactyly, n (%)	103 (83.1)	35 (85.4)	10 (83.3)	10 (90.9)	p = 0.912
Pitting scars or digital ulcers, n (%)	66 (53.2)	17 (41.5)	3 (25.0)	7 (63.6)	p = 0.140
Telangiectasia, n (%)	83 (74.1) (ND: 12)	29 (78.4) (ND: 4)	9 (100.0) (ND: 3)	9 (90.0) (ND: 1)	p = 0.238
Raynaud’s phenomenon, n (%)	124 (100.0)	40 (97.6)	11 (100.0) (ND: 1)	11 (100.0)	p = 0.313
Antibody profile, n (%)					p = 0.249
Anti-centromere Anti-topoisomerase I Anti-RNA polymerase III Other	74 (59.7) 10 (8.1) 1 (0.8) 39 (31.5)	8 (19.5) 8 (19.5) 0 (0.0) 25 (61.0)	8 (66.7) 1 (8.3) 1 (8.3) 2 (16.7)	3 (27.3) 2 (18.2) 2 (18.2) 4 (36.4)	
Calcinosis cutis, n (%)	40 (36.4) (ND: 14)	11 (32.4) (ND: 7)	4 (50.0) (ND: 4)	3 (50.0) (ND: 5)	p = 0.720
Gastrointestinal involvement, n (%)	88 (73.9) (ND: 5)	25 (62.5) (ND: 1)	6 (60.0) (ND: 2)	8 (72.7) (ND: 1)	p = 0.412
PFTs, median (IQR)					
% FVC % DLCO % Tiffeneau index	109.0 (95.5-120.0) (ND: 15) 74.0 (62.0-83.5) (ND: 15) 78.0 (70.0-82.0) (ND: 13)	94.5 (68.8-107.0)*** (ND: 3) 54.0 (42.5-70.5) (ND: 9) 80.0 (74.5-84.5) (ND: 4)	101 (83.8-116.8) 44.0 (42.0-61.0)* (ND: 3) 73.0 (67.3-80.8)	86.0 (65.0-107.0)*** 19.0 (17.3-35.5)** (ND: 12)79.0 (66.0-85.0)	p < 0.001 p < 0.001 p = 0.180
COPD, n (%)	28 (22.6)	6 (14.6)	3 (25.0)	3 (27.3)	p = 0.675
Medication, n (%)					
Immunosuppression Vasodilators Glucocorticoids	17 (13.7) 16 (12.9) 10 (8.1)	15 (36.6)** 3 (7.3) 7 (17.1)	0 (0.0) 9 (75.0)*** 0 (0.0)	5 (45.5)** 5 (45.5)** 3 (27.3)*	p = 0.001 p < 0.001 p = 0.065

SSc, systemic sclerosis; ILD, interstitial lung disease; PAH, pulmonary arterial hypertension; PFTs, pulmonary function tests; FVC, forced vital capacity; DLCO, diffusing capacity for carbon monoxide. *p<0.05, **p<0.01, ***p<0.001 vs no pulmonary involvement group (by Mann-Whitney U test). ND, not documented.

### Baseline comparison of sRAGE and HMGB1 levels

**Figure 1:**
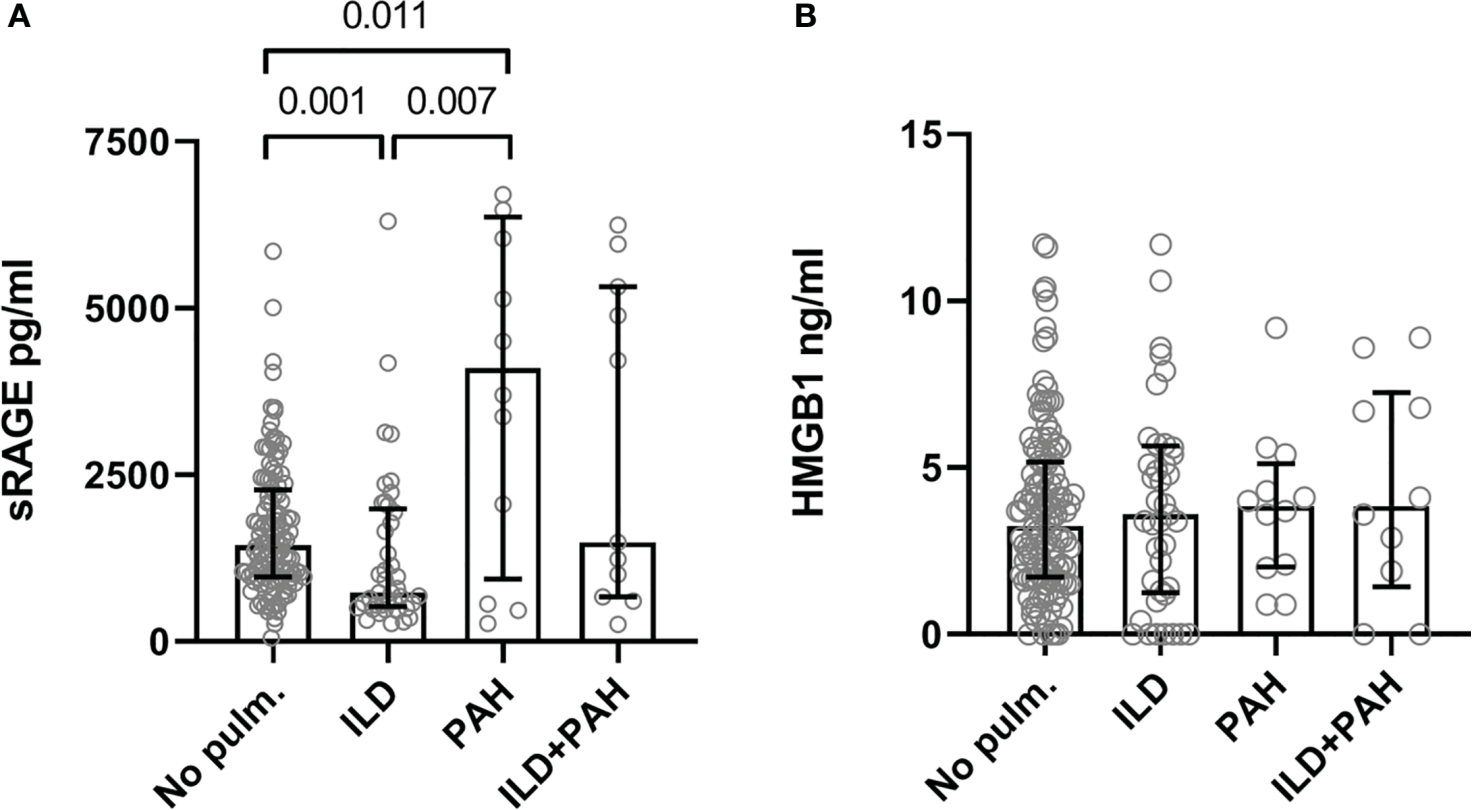
Serum levels of the sRAGE **(A)** and HMGB1 **(B)**. **(A)** Serum concentration levels are significantly elevated in SSc-PAH (PAH, N=12) patients compared to SSc-ILD (ILD, N=41) and SSc patients with no lung complications (No pulm., N=124). SSc-ILD patients had significantly lower concentration levels of serum sRAGE compared to SSc patients with no lung complications. SSc patients with PAH and ILD had serum sRAGE concentration levels comparable to SSc patients with no pulmonary complications. **(B)** Serum concentration levels of HMGB1 were comparable in all groups. Measurements are median values of sRAGE or HMGB1. sRAGE, soluble receptor for advanced glycation end products; HMGB1, high mobility group box 1; No pulm., no pulmonary involvement; ILD, interstitial lung disease; PAH, pulmonary arterial hypertension.

At baseline, levels of sRAGE were significantly higher in SSc-PAH patients (4099.0 pg/ml [936.3-6365.3], p = 0.011) but significantly lower in SSc-ILD (median 735.0 pg/ml [IQR 525.5-1988.5], p = 0.001) compared to patients without pulmonary involvement (1444.5 pg/ml [966.8-2276.0]). Levels of sRAGE in patients with both ILD and PAH (1487.0 pg/ml [670.0-5319.0]) were comparable to those without pulmonary involvement. No differences in HMGB1 levels were present between the different patient subgroups (No lung involvement: 3.3 [1.7-5.2]; ILD: 3.6 [1.3-5.7]; PAH: 3.9 [2.0-5.1]; ILD+PAH: 3.9 [1.4-7.3]) (**Figure 1**).

In the entire cohort, levels of sRAGE correlated positively with age (r = 0.218; p = 0.003), FVC (r = 0.242; p = 0.002), FEV1 (r = 0.254; p = 0.001) but not with DLCO (r = -0.023; p = 0.769). sRAGE levels were significantly higher in females compared to males (p = 0.002), also in patients who were positive for ACA (p < 0.001) compared to ACA negative patients, and in patients with puffy fingers or sclerodactyly (p = 0.039) compared to patients with no puffy fingers or sclerodactyly. sRAGE did not differ between lcSSc- and dcSSc patients, pitting scars or digital ulcers, telangiectasia, Raynaud’s phenomenon, calcinosis cutis, or intestinal involvement. HMGB1 levels were lower in patients with puffy fingers or sclerodactyly (p = 0.033) and higher in patients with telangiectasia (p = 0.027) compared to patients without puffy fingers or sclerodactyly, or telangiectasia.

**Table 2** shows the results of the regression analyses between sRAGE and important clinical determinants. The model showed that baseline sRAGE levels remained independently associated with PAH after adjustment for age, gender, ILD, COPD, ACA positivity, puffy fingers or sclerodactyly, use of vasodilators, and use of immunosuppression, antifibrotic therapy, or glucocorticoids. Noticeably, the presence of ACAs (p < 0.001) and puffy fingers or sclerodactyly (p = 0.017) were significantly associated with sRAGE levels at baseline (model 5).

**Table 2:** Multiple linear regression models: sRAGE and important clinical determinants.

	Model 1 (R^2^ = 0.172; p < 0.001) *St. ß (p = value)*	Model 2 (R^2^ = 0.188; p < 0.001) *St. ß (p = value)*	Model 3 (R^2^ = 0.261; p < 0.001) *St. ß (p = value)*	Model 4 (R^2^ = 0.284; p < 0.001) *St. ß (p = value)*	Model 5 (R^2^ = 0.287; p < 0.001) *St. ß (p = value)*
**PAH**	0.343 (< 0.001)	0.363 (< 0.001)	0.350 (< 0.001)	0.349 (< 0.001)	0.369 (< 0.001)
**Gender**	-0.142 (0.037)	-0.120 (0.081)	-0.048 (0.479)	-0.044 (0.508)	-0.046 (0.490)
**Age**	0.165 (0.016)	0.169 (0.016)	0.120 (0.078)	0.094 (0.168)	0.098 (0.153)
**ILD**		-0.128 (0.065)	-0.030 (0.664)	-0.026 (0.703)	-0.043 (0.557)
**COPD**		-0.023 (0.744)	-0.012 (0.854)	-0.008 (0.902)	-0.010 (0.879)
**Anti-centromere**			0.304 (< 0.001)	0.334 (< 0.001)	0.334 (< 0.001)
**Puffy finger or sclerodactyly**				0.155 (0.018)	0.158 (0.017)
**Use of vasodilators**					-0.040 (0.580)
**Use of immunosuppression, antifibrotic therapy, or glucocorticoids**					0.044 (0.511)

sRAGE, soluble receptor for advanced glycation end products; PAH, pulmonary arterial hypertension; ILD, interstitial lung disease; COPD, chronic obstructive pulmonary disease.

### Follow-up

**Figure 2:**
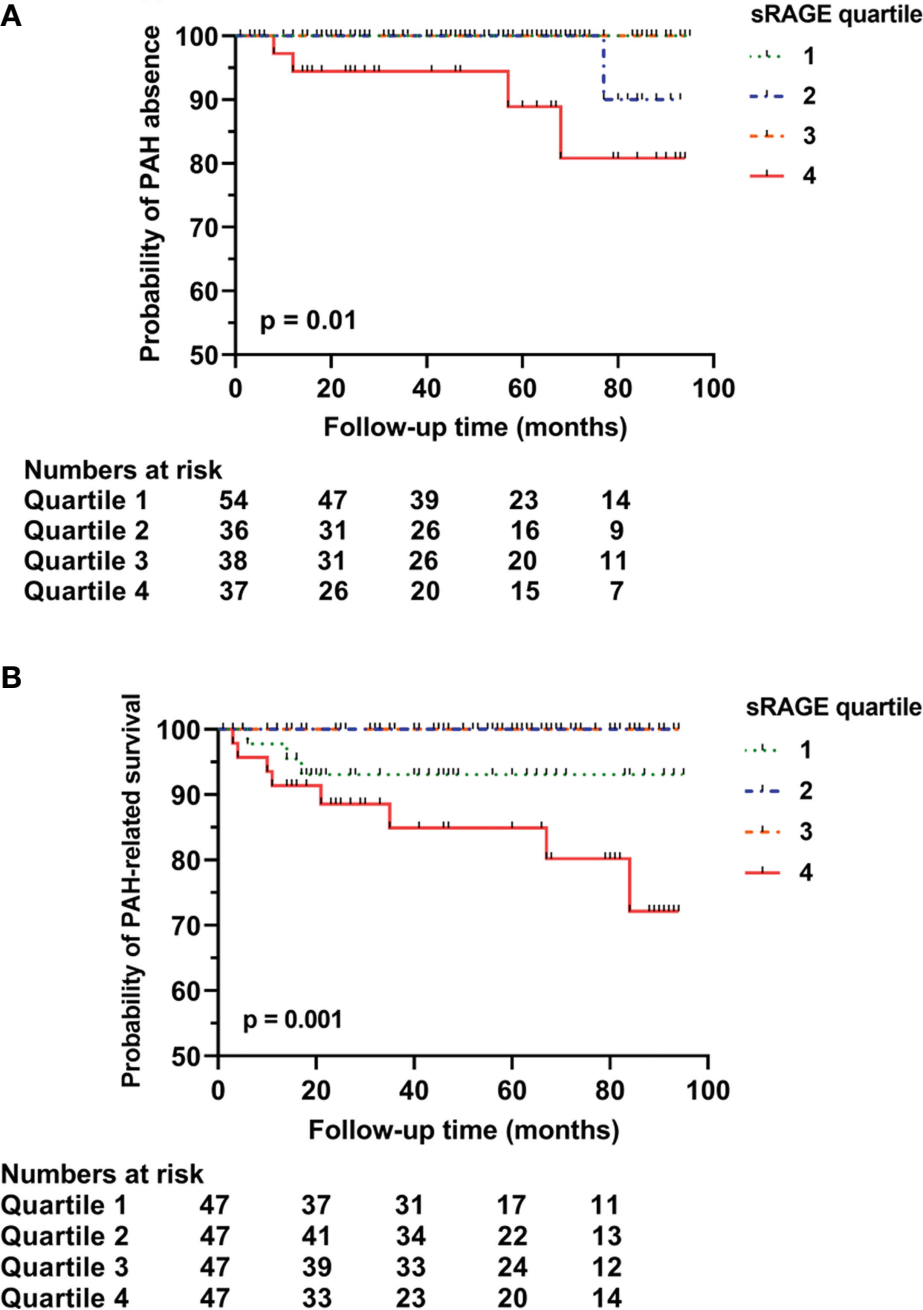
Predictive capacity of sRAGE for long-term pulmonary arterial hypertension rate **(A)**, and survival related to pulmonary arterial hypertension **(B)**. Survival curve of baseline sRAGE serum concentrations in SSc patients with no pulmonary involvement stratified into quartiles. **(A)** sRAGE concentration levels in the 4^th^ quartile significantly predicted the development of PAH in SSc patients (p=0.01). **(B)** sRAGE concentration levels in the 4^th^ quartile significantly predicted PAH-related mortality (p=0.001). sRAGE, soluble receptor for advanced glycation end products; PAH, pulmonary arterial hypertension.

Follow-up data of 124 patients without pulmonary involvement identified 11 (9%) patients with newly developed ILD and 5 (4%) patients with newly developed PAH (**Table 3**). The total median time of follow-up was 50 months (25-81 months); 56 months (33-82 months) for the development of ILD and 57 months (35-83 months) for the development of PAH. In the subcohort without PAH at baseline, sRAGE levels in the highest quartile (4^th^ quartile) predicted the development of PAH (p = 0.01) during follow-up (**Figure 2A**). Low sRAGE levels did not predict ILD development (p = 0.713). However, preliminary data from a small subset of 3 patients, in whom sequential samples were taken, showed that sRAGE levels decreased by approximately 250 pg/ml prior to ILD development. In this population, levels of HMGB1 did not predict PAH (p = 0.317) or ILD (p = 0.875).

**Table 3:** Patient characteristics at follow-up.

	SSc patients without pulmonary involvement at follow-up (n=109)	SSc patients with ILD (n=11)	SSc patients with PAH (n=5)	Kruskal Wallis Test
Age in years, median (IQR)	61 (54-71)	75 (60-80)*	75 (52-79)	p = 0.048
Female, n (%)	89 (81.7)	9 (81.8)	5 (100.0)	p = 0.769
Death, n (%)	6 (5.5)	1 (9.1)	1 (20.0)	p = 0.441
Extent of skin involvement, n (%)				p = 0.450
Limited Diffuse Other	101 (92.7) 8 (7.3) 0 (0.0)	9 (81.8) 1 (9.1) 1 (9.1)	5 (100.0) 0 (0.0) 0 (0.0)	
Follow-up duration in months, median (IQR)	N/A	56 (33-82)	57 (35-83)	p = 0.793
Puffy fingers or sclerodactyly, n (%)	90 (82.6)	11 (100.0)	3 (60.0)	p = 0.149
Pitting scars or digital ulcers, n (%)	57 (52.3)	6 (54.5)	3 (60.0)	p = 0.551
Telangiectasia, n (%)	72 (74.2) (ND: 12)	8 (72.7)	4 (80.0)	p = 0.933
Raynaud’s phenomenon, n (%)	109 (100.0)	11 (100.0)	5 (100.0)	p = 1.000
Antibody profile, n (%)				p = 0.627
Anti-centromere Anti-topoisomerase I Anti-RNA polymerase 3 Other	66 (60.6) 7 (6.4) 1 (0.9) 35 (32.2)	5 (45.5) 3 (27.3) 0 (0.0) 3 (27.3)	4 (80.0) 0 (0.0) 0 (0.0) 1 (20.0)	
Calcinosis cutis, n (%)	36 (37.9) (ND: 14)	3 (27.3)	1 (20.0)	p = 0.788
Gastrointestinal involvement, n (%)	74 (71.2) (ND: 5)	10 (90.9)	5 (100.0)	p = 0.322

SSc, systemic sclerosis; ILD, interstitial lung disease; PAH, pulmonary arterial hypertension; PFTs, pulmonary function tests; FVC, forced vital capacity; DLCO, diffusing capacity for carbon monoxide; N/A, not applicable. One patient had concomitantly ILD and PAH. *p<0.05 vs no pulmonary involvement group (by Mann-Whitney U test). ND, not documented.

### Mortality

Overall mortality was in 38% of the cases related to PAH, 17% to ILD, and 21% to other SSc-related complications including gastro-intestinal complications and progressive disease. During follow-up, in the entire cohort, sRAGE (p = 0.126) or HMGB1 (p = 0.404) were not predictive of overall mortality. However, a trend was seen for sRAGE levels in the 1^st^ and in 4^th^ quartiles to predict overall mortality (not shown). PAH-related mortality was significantly predicted by sRAGE levels in the highest quartile (4^th^ quartile) [p = 0.001 (**Figure 2B**)]. Mortality related to ILD was not predicted by low baseline sRAGE levels.

### Immunohistochemical expression of RAGE

**Figure 3:**
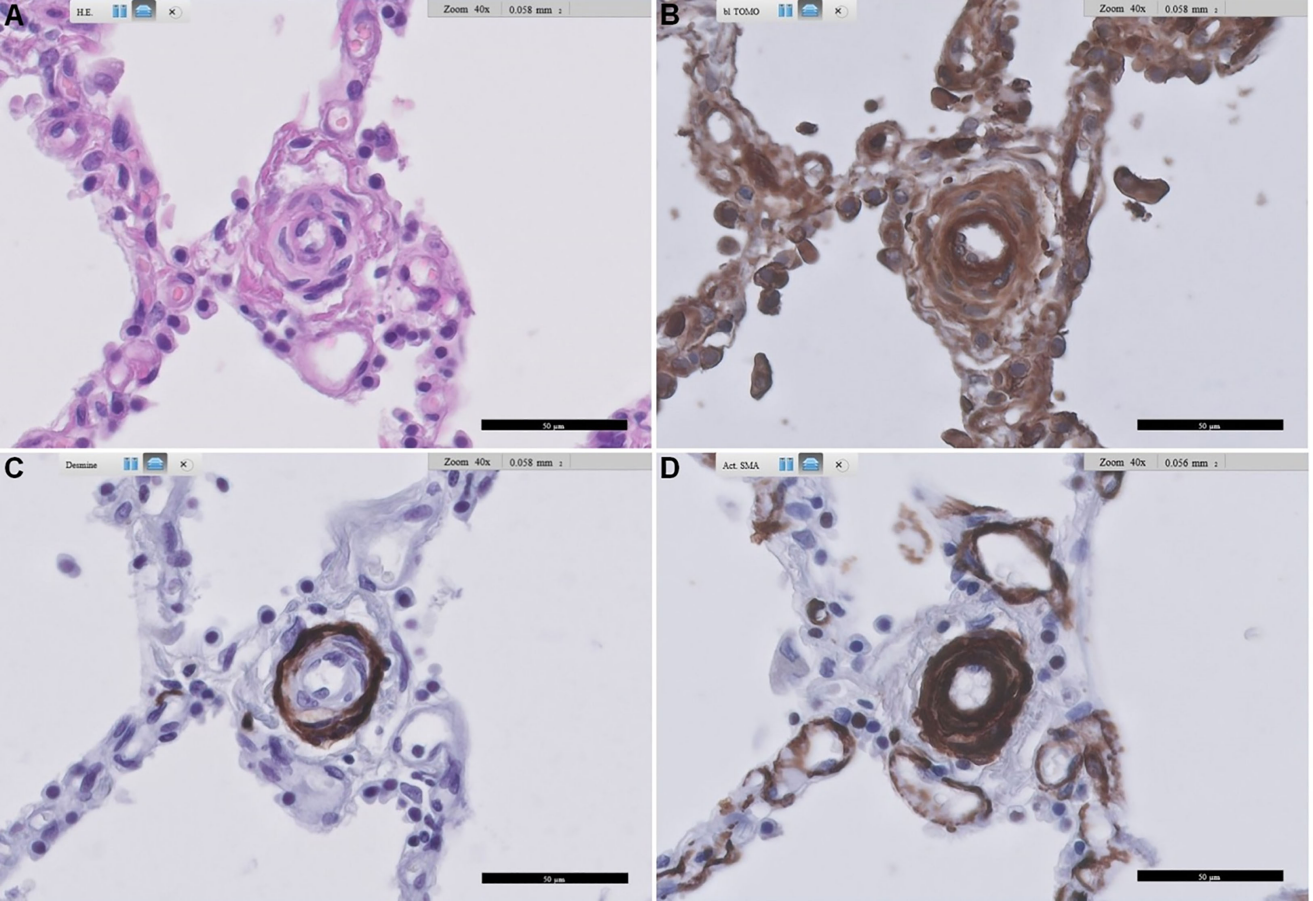
Representative images of RAGE staining in a primary pulmonary artery of a patient with SSc-related PAH. **(A)** shows standard Haematoxylin and Eosin-stained lung tissue, **(B)** shows RAGE staining, **(C)** shows desmin staining, and **(D)** shows alpha-smooth muscle actin staining. Arteriopathy in SSc is consistent with PAH with concentric thickening of the media and also concentric cellular increase in thickness of the intima. Scale bar, 50 µm. RAGE: receptor for advanced glycation end products; SSc: systemic sclerosis; PAH: pulmonary arterial hypertension.

**Figure 4:**
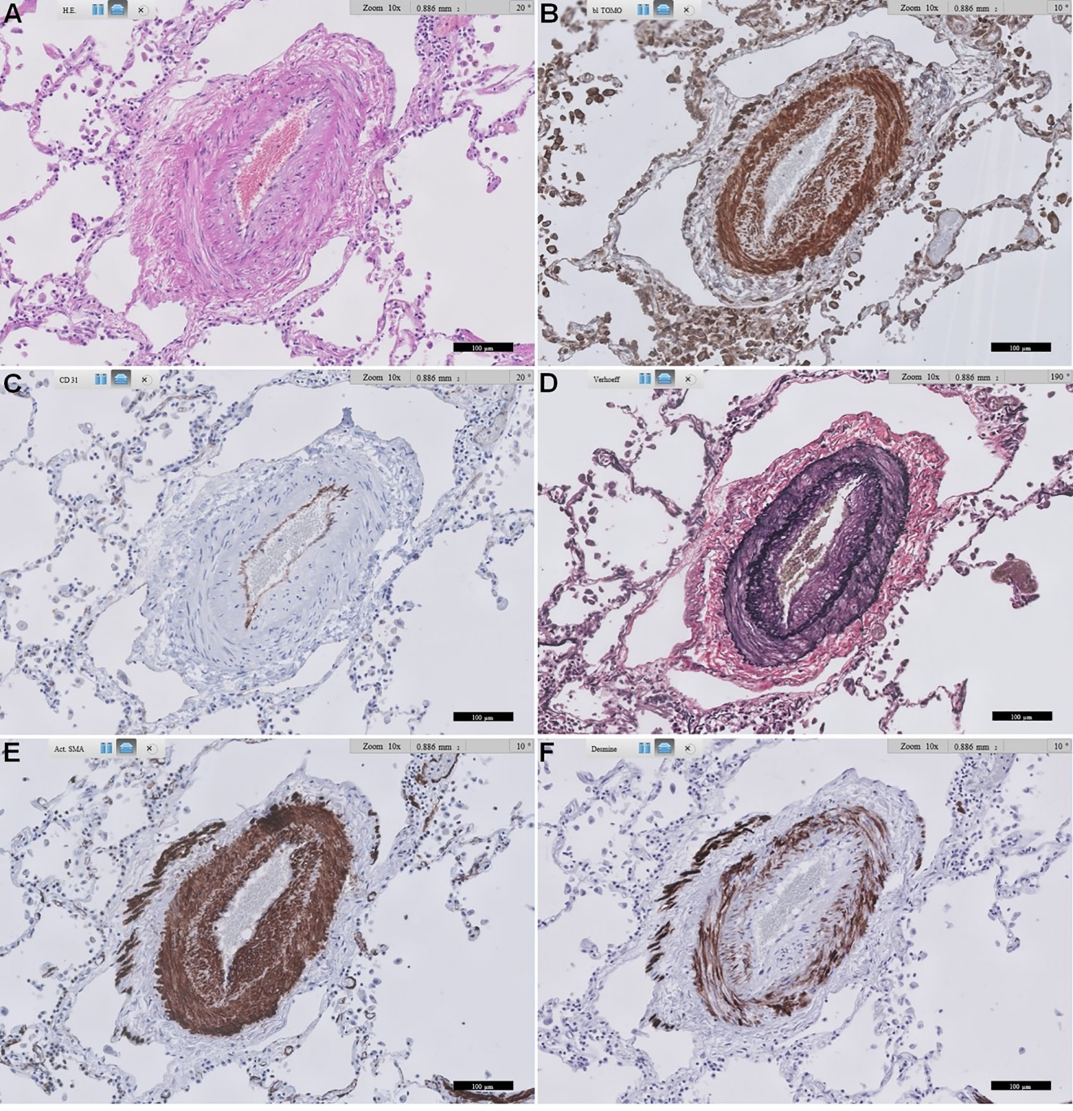
Representative images of RAGE staining in a secondary pulmonary artery of a patient with SSc-related PAH. **(A)** shows standard Haematoxylin and Eosin-stained lung tissue. **(B)** shows RAGE staining. **(C)** shows CD31 staining of endothelial cells. **(D)** shows Verhoeff’s elastin staining, delineating the inner and outer arterial elastic membrane, and some more diffusely stained elastin in intima and media. **(E)** shows α-smooth muscle actin staining of the media, and also quite extensively in the irregularly thickened intima, likely mainly myofibroblasts. **(F)** shows desmin staining, mainly smooth muscle cells in the media, and some in the intima. Scale bar, 100 µm. This representative image of a SSc-related PAH patient shows predominant expression of RAGE in smooth muscle cells in the media, followed by prominent expression in likely mainly myofibroblasts in the intima. RAGE: receptor for advanced glycation end products; SSc: systemic sclerosis; PAH: pulmonary arterial hypertension.

From our pilot immunohistochemical study, **Figures 3**, **4** show representative images of RAGE expression in primary lesions in small arterioles related to SSc vasculopathy, and in secondary lesions developed in due course in PAH in the pulmonary vasculature of SSc patients with PAH. The primary lesion (**Figure 3**) showed a clearly thickened media and a slightly thickened cellular intima, while the secondary lesion (**Figure 4**) showed clear, but irregular intima hyperplasia and a relatively normal surrounding media. In **Figure 3B**, expression of RAGE is seen most pronounced in the intima (inner layer; desmin negative), but also, somewhat less strong, in the thickened media, the surrounding, desmin positive layer. In **Figure 4B**, RAGE is located predominantly in the media and less in the intima. Comparing RAGE expression with desmin and α-SMA immunostains, the cells stained for RAGE are mainly smooth muscle cells in the media and are myofibroblasts in the intima. The endothelial layer appears negative for RAGE in the primary and secondary lesions. A summary of the IHC results is included in **Table 4**.

**Table 4:** Summary of IHC studies.

	Primary PAH lesion	Secondary PAH lesion
Media region	Clearly thickened	Normal
Intima region	Slightly thickened	Clear and irregular hyperplasia
RAGE positive staining	Intima (strong), Media (weak)	Media (strong) Intima (weak)
RAGE in smooth muscle cells	In Media (α-SMA and desmin positive)	
RAGE in myofibroblasts	In Intima (α-SMA positive and desmin negative)	
RAGE in endothelial cells	Absent	Absent

## Discussion

PAH and ILD are severe complications in SSc patients, and early detection is still a challenge. Since (s)RAGE acts as a major mediator in pulmonary disease, we investigated the capacity of sRAGE and its ligand, HMGB1 to predict future incidence of SSc-related pulmonary complications. This study shows that sRAGE is significantly higher in sera of patients with SSc-PAH, while it is significantly lower in patients with SSc-ILD, when compared to SSc patients without pulmonary disease. Moreover, we show that high baseline sRAGE levels could potentially be predictive for development of PAH and PAH-related mortality in SSc.

sRAGE has been implicated in various pathological processes in the lung (5, 10, 20). Iwamoto et al. (21) showed that sRAGE was lower in patients with COPD. Similarly, another study showed that levels of sRAGE were lower in patients with IPF and other types of ILD compared to control subjects (22). This is in line with our data as we found lower levels of sRAGE in SSc-ILD patients. In *in vitro* experiments, blocking of RAGE resulted in cell-matrix adhesion impairment, and RAGE knockdown led to an increase in cell proliferation and migration of alveolar epithelial cells and lung fibroblasts (23). This implies that loss of RAGE can lead to fibrotic changes in lung tissues, of which ILD/IPF are important examples. Although in our current study we were not able to demonstrate a predictive value of low sRAGE levels for SSc-ILD, we did find in a small subset of patients (N=3) in whom sequential samples were taken that sRAGE decreased prior to ILD development. This is in line with the correlation of sRAGE with FVC, but not with DLCO, potentially indicating the decrease in sRAGE occurs in more advanced stages of ILD. This observation needs validation in a larger cohort with sequential measurements.

Recently, a group of scientists ran a panel of eight proteins to find a proteomic biomarker signature using a machine-learning approach to distinguish SSc patients with and without PAH (24). Serum samples of 77 patients with PAH and 80 patients without PAH underwent proteomic screening using a multiplex immunoassay. Among all proteins, RAGE turned out to be the most prominent protein to identify SSc-PAH patients. Moreover, a recent study by Nakamura et al. (25) demonstrated the potential role of RAGE in the proliferation of pulmonary artery smooth muscle cells in patients with PAH. This might implicate the pathogenetic role of RAGE and clarify its upregulation in sera of SSc-PAH patients. While these findings confirm the importance of RAGE in the classification and pathogenesis of PAH, our follow-up findings show that high baseline sRAGE levels may also be used to identify SSc patients who will develop PAH. Our results also show that sRAGE may be used to predict PAH-related mortality in SSc.

Our regression analysis shows that ACA presence is significantly correlated with sRAGE levels. Several studies have associated the presence of ACA with development and progression of PAH but could not define whether ACA has pathogenetic effects leading to development of PAH (26, 27). There is indeed a growing body of evidence that immune complexes of SSc-related autoantibodies have pathogenetic effects on skin fibroblasts and endothelial cells. However, to our knowledge, no study has investigated whether these effects lead to PAH development. Finding the primary pathogenetic factor, or possible RAGE-ACA interactions, should be subject to further studies.

In general, HMGB1 is released after cell injury. However, while some studies report increased HMGB1 levels in PAH and IPF, others report no differences between patients and controls (28, 29). We showed that levels of HMGB1 were not different between the subsets in our study and neither predicted pulmonary events nor mortality. This is in accordance with a previous study showing that sRAGE, but not HMGB1, was higher in patients with pulmonary hypertension, especially in PAH and chronic thromboembolic pulmonary hypertension (28). Based on this, we conclude that HMGB1 may not be an appropriate marker for pulmonary involvement and survival in SSc. This may implicate that the upregulation of RAGE in SSc lung tissue is not related to HMGB1 but is possibly due to other RAGE ligands. These include other DAMPs (AGEs and/or calgranulins) which have previously been shown to be strongly associated with vascular disease and arterial wall remodeling and are increased in PAH (14, 30).

Taken together, it is plausible that the HMGB1 (or other DAMPs) and ACA-autoantibodies together are the actual pathogenetic factor forming immune complexes binding to RAGE in the lung and leading to the development of PAH in SSc patients. And the high sRAGE levels are a coping mechanism (decoy receptor) working to attenuate the overstimulated DAMPs-RAGE axis which, for unknown reasons, does not work in patients developing PAH. Page et al. (31) have demonstrated that co-stimulation of peripheral blood mononuclear cells (PBMCs, regulators of the immune system) with calgranulin leads to increased levels of proinflammatory cytokines (in a RAGE-dependent manner) in ANCA-associated vasculitides (AAV), a group of systemic autoimmune diseases with specific phenotypes. They also reported that DAMPs levels in the circulation vary according to the activity/subtype of the disease. Similarly, sRAGE levels were comparable except for active AAV patients where sRAGE levels were significantly lower. The reasons for these differences are unknown but the authors claim that this is due to disease-specific differences. Our data add considerable strength to what the authors reported by showing distinct levels of sRAGE in different disease subtypes.

Patients with IPF had worse survival rates after a 3-year follow-up when sRAGE levels were low at baseline (32). In our study, low sRAGE levels did not significantly show decreased ILD-related survival. This may have been due to that our study is underpowered, or it might be because while SSc-ILD and IPF pathogeneses could share some similarities, they are two distinct diseases. Importantly, SSc-ILD differs from IPF as it is a fibro-inflammatory disease complication, while the latter is predominantly fibrotic which might allow a divergent profile of sRAGE between both diseases (33).

Different therapies, like nifedipine, have the ability to downregulate the expression of RAGE (34). We were not able to investigate the association between sRAGE levels and calcium antagonists because no patient with SSc-PAH in our cohort used this type of medication. Nakamura et al. (25) reported that AS-1, an inhibitor of RAGE signalling, suppressed the proliferation of smooth muscle cells of patients with idiopathic and heritable PAH, showing that inhibition of RAGE signalling may be a new therapeutic target for PAH. Also, a recent study demonstrated inhibitory effects of a short and single-stranded DNA directed against RAGE (RAGE aptamer) on the development of PAH in rats (35). This addresses a new therapeutic option for PAH which should be investigated in further studies.

Our pilot immunohistochemical data show expression of RAGE in the thickened intima of the primary arterial lesion related to SSc vasculopathy while the endothelium appears to be negative. Secondary arterial lesions developed in due course in PAH show also clear RAGE staining in the thickened intima. The thickening of the intima in both lesions may be due to infiltration by myofibroblasts since the intima is desmin negative and grossly α-SMA positive. Additionally, the media of both vascular lesions is clearly positive for RAGE, which has been reported before in other forms of PAH (25). Although speculative on these limited pilot data, we hypothesize that the higher systemic RAGE levels in SSc patients who develop PAH are associated with higher RAGE expression in the intima. Possibly, the absence of RAGE staining on endothelial cells might indicate shedding of RAGE into circulation. Additionally, we cannot rule out the possibility that smooth muscle cell proliferation in the media could contribute to a certain extent to the development of other forms of pulmonary hypertension.

This study has some limitations. First, PAH is defined as mPAP > 20 mmHg in the most recent guideline of PAH (36). In this study, the 2015 ESC/ERS guideline for the diagnosis of PAH by Galiè et al. (20) was used to define PAH as mPAP > 25 mmHg as it was practised at our center. This may have resulted in missing patients. Second, measures of diagnostic accuracy have not been calculated due to the limited sample size of affected patients. Also, as a consequence, Cox regression and prediction of mortality in the subset of patients with PAH could not be calculated. Preferably, these measurements should be conducted in future, prospective larger studies and correction for potential confounders need to be performed. Third, the smoking status of the patients was not analyzed, which may have influenced levels of sRAGE. Fourth, we could not check the values of other ligands of RAGE such as AGEs or s100 proteins. Finally, due to the limited availability of PAH lung tissue sections, our immunohistochemistry studies left us with cautious conclusions regarding RAGE source and location. These preliminary data need further exploration in larger series, and this hypothesis needs to be confirmed in experimental studies.

Of importance, our data may be useful to design prospective, longitudinal, multi-center studies on early biomarkers in pulmonary involvement and mortality in SSc to improve and strengthen our observations. As mentioned above, preliminary data of sRAGE values in consecutive blood samples of a few patients with SSc showed a clear drop in sRAGE before the development of ILD. Therefore, it would be essential to repeat sRAGE measurements in longitudinal samples of SSc patients to investigate whether sRAGE levels follow the disease course on- and off-treatment, in order to qualify as a biomarker for SSc lung complications.

Nowadays, screening for PAH is performed by transthoracic echocardiogram and diagnosed by right heart catheterisation (20, 36). Our study stresses the importance of further investigations to establish this novel predictive “non-invasive” biomarker for the new onset of PAH in SSc. Ultimately if our results are replicated independently, this could mean that sRAGE levels may be used as a simple blood test to differentiate between low-risk and high-risk patients prone to developing PAH in practice, consequently with the possibility of earlier referral of high-risk patients to a cardiologist.

In conclusion, we show that high sRAGE levels, but not HMGB1, in patients with SSc at baseline may be used to predict new onset of PAH related to SSc. Moreover, high sRAGE levels may predict lower survival rates due to PAH in SSc. Future research should focus on including a larger cohort to correct for confounding factors and finding a cut-off value to calculate diagnostic accuracy. Finally, our findings suggest that sRAGE may function as a disease biomarker that identifies development of SSc-PAH and allows early intervention.

## Data availability statement

The raw data supporting the conclusions of this article will be made available by the authors, without undue reservation.

## Ethics statement

Ethical review and approval was not required for the study on human participants in accordance with the local legislation and institutional requirements. Written informed consent for participation was not required for this study in accordance with the national legislation and the institutional requirements.

## Author contributions

Conceptualization and methodology, IA and DM; Formal Analysis, IA; Investigation, IA; Resources, YA-A, AS, BD-v, CR, HG, MD, CG, WT; Data Curation, IA, YA-A; Writing – Original Draft Preparation, IA; Writing – Review & Editing, AJS, AS, BD-v, CR, HG, JW, MD, CG, YA-A, WT, and DM; Visualization, IA; Supervision, AJS, JW, and DM; Project Administration, IA, YA-A. All authors contributed to the article and approved the submitted version. 

## References

[1] Tyndall AJ Bannert B Vonk M Airò P Cozzi F Carreira PE. Causes and risk factors for death in systemic sclerosis: a study from the EULAR scleroderma trials and research (EUSTAR) database. Ann Rheum Dis (2010) 69(10):1809–15. doi: 10.1136/ard.2009.114264 20551155

[2] Bonhomme O André B Gester F de Seny D Moermans C Struman I. Biomarkers in systemic sclerosis-associated interstitial lung disease: review of the literature. Rheumatology (2019) 58(9):1534–46. doi: 10.1093/rheumatology/kez230 PMC6736409 31292645

[3] Hickey PM Lawrie A Condliffe R. Circulating protein biomarkers in systemic sclerosis related pulmonary arterial hypertension: a review of published data. Front Med (Lausanne) (2018) 5(6):1–7. doi: 10.3389/fmed.2018.00175 29928643 PMC5997816

[4] Diekmann F Chouvarine P Sallmon H Meyer-Kobbe L Kieslich M Plouffe BD. Soluble receptor for advanced glycation end products (sRAGE) is a sensitive biomarker in human pulmonary arterial hypertension. Int J Mol Sci (2021) 22(16):8591. https://www.mdpi.com/1422-0067/22/16/8591. doi: 10.3390/ijms22168591 34445297 PMC8395319

[5] Oczypok EA Perkins TN Oury TD. All the “RAGE” in lung disease: the receptor for advanced glycation endproducts (RAGE) is a major mediator of pulmonary inflammatory responses. Paediatr Respir Rev (2017) 23:40–9. doi: 10.1016/j.prrv.2017.03.012 PMC5509466 28416135

[6] Schmidt AM Du YS Yan SF Stern DM. The multiligand receptor RAGE as a progression factor amplifying immune and inflammatory responses. J Clin Invest (2001) 108(7):949–55. doi: 10.1172/JCI200114002 PMC200958 11581294

[7] Nienhuis HLA Westra J Smit AJ Limburg PC Kallenberg CGM Bijl M. AGE and their receptor RAGE in systemic autoimmune diseases: an inflammation propagating factor contributing to accelerated atherosclerosis. Autoimmunity (2009) 42(4):302–4. doi: 10.1080/08916930902831746 19811283

[8] Hudson BI Carter AM Harja E Kalea AZ Arriero M Yang H. Identification, classification, and expression of RAGE gene splice variants. FASEB J (2008) 22(5):1572–80. doi: 10.1096/fj.07-9909com 18089847

[9] Zhang L Bukulin M Kojro E Roth A Metz VV Fahrenholz F. Receptor for advanced glycation end products is subjected to protein ectodomain shedding by metalloproteinases. J Biol Chem (2008) 283(51):35507–16. doi: 10.1074/jbc.M806948200 18952609

[10] Buckley ST Ehrhardt C. The receptor for advanced glycation end products (RAGE) and the lung. J BioMed Biotechnol (2010) 2010:917108. doi: 10.1155/2010/917108 20145712 PMC2817378

[11] Mukherjee TK Mukhopadhyay S Hoidal JR. Implication of receptor for advanced glycation end product (RAGE) in pulmonary health and pathophysiology. Respir Physiol Neurobiol (2008) 162(3):210–5. doi: 10.1016/j.resp.2008.07.001 18674642

[12] Miniati M Monti S Basta G Cocci F Fornai E Bottai M. Soluble receptor for advanced glycation end products in COPD: relationship with emphysema and chronic cor pulmonale: a case-control study. Respir Res (2011) 12:37. doi: 10.1186/1465-9921-12-37 21450080 PMC3072955

[13] Meloche J Courchesne A Barrier M Carter S Bisserier M Paulin R. Critical role for the advanced glycation end-products receptor in pulmonary arterial hypertension etiology. J Am Heart Assoc (2013) 2(1):1–14. doi: 10.1161/JAHA.112.005157 PMC3603259 23525442

[14] Jia D He Y Zhu Q Liu H Zuo C Chen G. RAGE-mediated extracellular matrix proteins accumulation exacerbates HySu-induced pulmonary hypertension. Cardiovasc Res (2017) 113(6):586–97. doi: 10.1093/cvr/cvx051 28407046

[15] Watanabe H Son M. The immune tolerance role of the hmgb1-rage axis. Cells (2021) Vol. 10:1–14. doi: 10.3390/cells10030564 PMC8001022 33807604

[16] Bauer EM Shapiro R Zheng H Ahmad F Ishizawar D Comhair SA. High mobility group box 1 contributes to the pathogenesis of experimental pulmonary hypertension *via* activation of toll-like receptor 4. Mol Med (2012) 18(12):1509–18. doi: 10.2119/molmed.2012.00283 PMC3576475 23269975

[17] Zheng JN Li Y Yan YM Yu Y Shao WQ Wang Q. Increased serum calpain activity is associated with HMGB1 levels in systemic sclerosis. Arthritis Res Ther (2020) 22(1):110. doi: 10.1186/s13075-020-02195-y 32393322 PMC7216546

[18] van den Hoogen F Khanna D Fransen J Johnson SR Baron M Tyndall A. 2013 classification criteria for systemic sclerosis: an American college of Rheumatology/European league against rheumatism collaborative initiative. Arthritis Rheum (2013) 65(11):2737–47. doi: 10.1002/art.38098 PMC3930146 24122180

[19] Wells AU Hirani N. Interstitial lung disease guideline. Thorax (2008) 63(Supplement 5):v1–58. doi: 10.1136/thx.2008.101691 18757459

[20] Galiè N Humbert M Vachiery JL Gibbs S Lang I Torbicki A. ESC/ERS guidelines for the diagnosis and treatment of pulmonary hypertension. Eur Heart J (2015) 37(1):67–119. doi: 10.1093/eurheartj/ehv317 26320113

[21] Iwamoto H Gao J Pulkkinen V Toljamo T Nieminen P Mazur W. Soluble receptor for advanced glycation end-products and progression of airway disease. BMC Pulm Med (2014) 14(1):1–7. doi: 10.1186/1471-2466-14-68 24758342 PMC4021457

[22] Manichaikul A Sun L Borczuk AC Onengut-Gumuscu S Farber EA Mathai SK. Plasma soluble receptor for advanced glycation end products in idiopathic pulmonary fibrosis. Ann Am Thorac Soc (2017) 14(5):628–35. doi: 10.1513/AnnalsATS.201606-485OC PMC5427736 28248552

[23] Queisser MA Kouri FM Königshoff M Wygrecka M Schubert U Eickelberg O. Loss of RAGE in pulmonary fibrosis: molecular relations to functional changes in pulmonary cell types. Am J Respir Cell Mol Biol (2008) 39(3):337–45. doi: 10.1165/rcmb.2007-0244OC 18421017

[24] Bauer Y de Bernard S Hickey P Ballard K Cruz J Cornelisse P. Identifying early pulmonary arterial hypertension biomarkers in systemic sclerosis: machine learning on proteomics from the DETECT cohort. Eur Respir J (2021) 57(6):2002591. doi: 10.1183/13993003.02591-2020 33334933 PMC8276065

[25] Nakamura K Sakaguchi M Matsubara H Akagi S Sarashina T Ejiri K. Crucial role of RAGE in inappropriate increase of smooth muscle cells from patients with pulmonary arterial hypertension. PloS One (2018) 13(9):e0203046. doi: 10.1371/journal.pone.0203046 30180189 PMC6122782

[26] van Leeuwen NM Wortel CM Fehres CM Bakker JA Scherer HU Toes REM. Association between centromere- and topoisomerase-specific immune responses and the degree of microangiopathy in systemic sclerosis. J Rheumatol (2021) 48(3):402–9. doi: 10.3899/jrheum.191331 32482649

[27] Kampolis C Plastiras SC Vlachoyiannopoulos PG Moyssakis I Tzelepis GE. The presence of anti‐centromere antibodies may predict progression of estimated pulmonary arterial systolic pressure in systemic sclerosis. Scand J Rheumatol (2008) 37(4):278–83. doi: 10.1080/03009740801978871 18612928

[28] Suzuki S Nakazato K Sugimoto K Yoshihisa A Yamaki T Kunii H. Plasma levels of receptor for advanced glycation end-products and high-mobility group box 1 in patients with pulmonary hypertension. Int Heart J (2016) 57(2):234–40. doi: 10.1536/ihj.15-188 26973260

[29] Hamada N Maeyama T Kawaguchi T Yoshimi M Fukumoto J Yamada M.

[30] Farmer DGS Kennedy S. RAGE, vascular tone and vascular disease. Pharmacol Ther (2009) 124(2):185–94. doi: 10.1016/j.pharmthera.2009.06.013 19616578

[31] Page TH Chiappo D Brunini F Garnica J Blackburn J Dudhiya F. Danger-associated molecular pattern molecules and the receptor for advanced glycation end products enhance ANCA-induced responses. Rheumatol (United Kingdom) (2022) 61(2):834–45. doi: 10.1093/rheumatology/keab413 PMC8824420 33974049

[32] Machahua C Montes-Worboys A Planas-Cerezales L Buendia-Flores R Molina-Molina M Vicens-Zygmunt V. Serum AGE/RAGEs as potential biomarker in idiopathic pulmonary fibrosis. Respir Res (2018) 19(1):1–9. doi: 10.1186/s12931-018-0924-7 30409203 PMC6225674

[33] Herzog EL Mathur A Tager AM Feghali-Bostwick C Schneider F Varga J. Review: interstitial lung disease associated with systemic sclerosis and idiopathic pulmonary fibrosis: how similar and distinct? vol. 66, arthritis and rheumatology. John Wiley Sons Inc (2014) 66:1967–78. doi: 10.1002/art.38702 PMC4340472 24838199

[34] Prasad K. AGE-RAGE stress in the pathophysiology of pulmonary hypertension and its treatment. Int J Angiology (2019) 28(2):71–9. doi: 10.1055/s-0039-1687818 PMC6679961 31384104

[35] Nakamura K Akagi S Ejiri K Yoshida M Miyoshi T Sakaguchi M. Inhibitory effects of RAGE-aptamer on development of monocrotaline-induced pulmonary arterial hypertension in rats. J Cardiol (2021) 78(1):12–6. doi: 10.1016/j.jjcc.2020.12.009 33386219

[36] Simonneau G Montani D Celermajer DS Denton CP Gatzoulis MA Krowka M. Haemodynamic definitions and updated clinical classification of pulmonary hypertension. Eur Respir J (2019) 53(1):1801913. doi: 10.1183/13993003.01913-2018 30545968 PMC6351336

